# About Bees

**Published:** 1879-10

**Authors:** 


### MISCELLANEOUS.

About Bees.—The king is stingless, "armed only with his majestic port." Modern science regards this so-called king as mother and monarch of the hive. It is found that she lives four years, and is hatched from the egg in fourteen days, while the workers require twenty-one days, and the drones twenty-four. These strange figures are part of the mystery attaching to bees; but a still more curious fact connected with this point is that the bees have the power at will of developing common eggs into queen bees. This is done by removing an egg into a royal cell, and feeding the little grub with a substance of a milky, gelatinous appearance known as "royal jelly." These facts have been ascertained without doubt by Mr. Pittigrew, one of the most successful bee keepers of the day; though what the exact analysis of this "royal jelly" may be is utterly unknown. The chief function of the queen in the hive is to lay eggs, from which the future population will spring. A healthy queen during her life is estimated to lay the enormous number of 800,000. Often in the heat of summer, for two months together, she will lay 2,000 a day. Whether these eggs are all alike, or whether some are distinctly worker eggs, is one of the numerous questions on which all bee keepers are at issue. The working bees form the life and prosperity of the hive. To them belong industry, labor, patience, ingenuity—in short, all the virtues of the race; and while each knows his own duty, and does it, the efforts of all are directed toward the weal of the community. The working bee never lives longer than nine months; they labor so incessantly that it is supposed they never sleep. The daily consumption and waste of a large hive of bees in summer may be taken at two pounds of honey; it will show the industry of the working bees to bear in mind that beyond this, such a hive in favorable weather will often accumulate honey to the amount of four and six pounds daily. Indeed, it is upon record that a hive once gained twenty pounds weight of it in two days. It is curious that even a wild hive of bees can soon be taught to recognize and refrain from attacking people who approach them. No wonder that the ancients esteemed them divine; that their poet laureate, according to the Platonic philosophy, assigns them "a participation in the supreme mind and the heavenly influences," and that another speaks of their powers of presaging wind and fine weather.

